# Malignant transformation of deep infiltrating endometriosis at the uterosacral ligament to endometrioid adenocarcinoma: a rare Case Report

**DOI:** 10.3389/fonc.2026.1837994

**Published:** 2026-10-14

**Authors:** Boyi Ma, Jia Xi, Chenlu Ren, Ziting Chen, Wei Zhang, Hong Yang, Shuhua Zhao

**Affiliations:** 1 Department of Obstetrics and Gynecology, Xijing Hospital, The Fourth Military Medical University, Xi’an, Shaanxi, China; 2 Graduate Student Affairs Office, Xi’an Medical University, Xi’an, Shaanxi, China; 3 Honghui Hospital, Xi’an Jiaotong University, Xi’an, Shaanxi, China; 4 School of Medicine, Northwest University, Xi’an, China

**Keywords:** case report, Deep infiltrating endometriosis, Endometrioid adenocarcinoma, malignant transformation, Uterosacral ligament

## Abstract

**Background:**

The malignant transformation of endometriosis has garnered widespread attention; however, most cases of malignant transformation occur in ovarian endometriomas, while extraovarian malignant transformation is rare. We present an uncommon instance of extraovarian endometrioid adenocarcinoma originating from the uterosacral ligament(USL) in a 41-year-old female, which emerged from deep infiltrating endometriosis of the uterosacral ligament 4 years after a previous clinical diagnosis of adenomyosis.

**Case presentation:**

A 41-year-old woman, presenting with progressively worsening secondary dysmenorrhea for 4 years and dyspareunia for over 1 year, underwent “laparoscopic total hysterectomy, left adnexectomy, right salpingectomy, right ovarian endometrioma cystectomy, bilateral uterosacral ligament DIE lesion resection, anterior rectal resection, rectal DIE lesion resection with end-to-end anastomosis, left ureteral DIE lesion excision, and pelvic adhesiolysis.” Postoperative pathology revealed a well-differentiated endometrioid adenocarcinoma of the uterosacral ligament (FIGO grade 1), arising from malignant transformation of pelvic endometriosis. Subsequently, a completion surgery was performed, consisting of “transabdominal right oophorectomy, pelvic and para-aortic lymphadenectomy, omentectomy, biopsy of an ileal surface lesion, resection of a right pelvic wall peritoneal lesion, and pelvic adhesiolysis.” Postoperative pathological examination revealed a metastatic carcinoma in the right obturator lymph node. The final diagnosis was stage IV according to the FIGO 2009 staging system (FIGO stage IV) pelvic endometriosis-associated malignancy with direct invasion of the rectal wall by the USL adenocarcinoma. The patient underwent six cycles of adjuvant chemotherapy with paclitaxel and carboplatin given every three weeks. Over five years after finishing treatment, there are no signs of recurrence.

**Conclusion:**

This paper describes an unusual case where deep infiltrating endometriosis of the uterosacral ligament led to malignant transformation. During long-term treatment and surgery for endometriosis, the potential for malignant transformation and the malignant characteristics of endometriosis should be fully considered. Prompt and thorough surgical intervention is crucial.

## 1 Introduction

Endometriosis is a common benign condition in women’s health. Deep infiltrating endometriosis (DIE) is a unique form where lesions can reach depths greater than 5 mm, involving several pelvic organs (1). Among all endometriosis cases, 20% involve deep infiltrating endometriosis, with lesions primarily found in the intestine, especially in the rectum, appendix, and ileum (2). Although most endometriotic lesions are benign, approximately 0.5% to 1% of cases may undergo malignant transformation. Ovarian endometriomas are the primary source of most malignant tumors that arise from endometriosis (3). Nevertheless, it is uncommon for malignant tumors to develop from superficial peritoneal endometriosis or deep infiltrating endometriosis lesions, and a standard treatment has not been determined. Herein, we report a rare case of endometrioid adenocarcinoma arising from malignant transformation of ectopic endometrium in the uterosacral ligament, which evolved from pelvic deep infiltrating endometriosis of the uterosacral ligament, four years after a previous clinical diagnosis of adenomyosis.

## 2 Case presentation

A 41-year-old Chinese woman, who has been pregnant twice and has had two cesarean deliveries, reported experiencing increasingly severe menstrual abdominal pain that began 4 years ago. At 13, she experienced menarche and maintained a regular menstrual cycle, and had a history of dysmenorrhea. At the age of 37, she developed intolerable, progressively worsening dysmenorrhea and was diagnosed with adenomyosis. During that period, she was treated with oral traditional Chinese medicine (specific details unknown), which alleviated her symptoms. The original imaging findings and diagnostic criteria for the external-hospital diagnosis of adenomyosis were not available; this history is reported only as a temporal reference and does not imply a causal relationship between the prior treatment and the subsequent malignant transformation. At the age of 40, she developed severe dysmenorrhea, anal bulging pain, and dyspareunia, which were slightly relieved by oral administration of the analgesic “Ibuprofen”. However, six months later, the cyclical dysmenorrhea transformed into persistent, unrelieved pain in the lower abdomen and lumbosacral region, accompanied by elevated blood pressure. She underwent diagnostic laparoscopy at another hospital and was diagnosed with stage IV pelvic endometriosis (without surgical resection of the lesions;rASRM stage IV endometriosis). Colposcopy revealed patchy, dark red nodular protrusions in the posterior vaginal fornix, with an approximately 1 cm ulcerated surface on the lesion (**Supplementary Figures A–C**). Transvaginal ultrasound (**Supplementary Figures 2A, B**) and pelvic magnetic resonance imaging (**Figures 1A–E**) demonstrated extensive DIE lesions involving multiple anatomical compartments: the posterior uterine wall, rectovaginal septum/uterosacral ligament, and posterior to the right ovary. The lesions were closely adherent to the rectum and sigmoid colon with restricted mobility. MRI further demonstrated the lesion encasing the distal left ureter with resultant luminal narrowing (**Figure 1C**), leading to secondary dilatation and hydronephrosis of the left ureter and renal pelvis (**Figure 1E**), which was confirmed by the radionuclide renogram (**Supplementary Figure 3**) showing severe left renal functional impairment. Endoscopic ultrasonography (**Supplementary Figures 4A, B**) demonstrated a hypoechoic lesion adjacent to the rectum infiltrating the muscularis propria of the bowel wall. A radionuclide renogram (**Supplementary Figure 3**) revealed severe functional impairment of the left kidney. Endoscopic ultrasonography (**Supplementary Figures 4A, B**) revealed a hypoechoic lesion adjacent to the rectum, infiltrating the muscularis propria. During laparoscopic surgery for endometriosis, the following findings were observed: a DIE lesion in the left uterosacral ligament penetrating the vaginal wall, which was significantly thickened, hardened(**Figures 2A–D**), and densely adherent to the rectum and sigmoid colon; a rectal lesion infiltrating the full thickness of the intestinal wall (**Figures 2E, F**); and a thickened, hardened segment of the left ureter in the pelvic portion, approximately 2 cm in length, with no visible peristalsis, densely adherent to the surrounding tissues. As the patient did not desire to preserve fertility, a semi-radical surgical procedure was performed: laparoscopic total hysterectomy, left adnexectomy, right salpingectomy, right ovarian endometrioma cystectomy, bilateral uterosacral ligament DIE lesion resection, anterior rectal resection, rectal DIE lesion resection, end-to-end intestinal anastomosis, left ureteral DIE lesion excision, and pelvic adhesiolysis. The patient received routine step-down therapy postoperatively (GnRH-a for 6 months), recovered well, and was discharged on the 5th postoperative day. However, the pathological results (**Figures 3A, B**) revealed diffuse proliferation of atypical glands in the uterus, cervix, and vagina, with infiltration into the smooth muscle. Atypical glands were observed in the ectopic lesions at the uterosacral ligament. Immunohistochemical staining suggested a well-differentiated endometrioid adenocarcinoma (FIGO grade 1) with perineural invasion. Pathological examination of the rectal resection specimen confirmed that the USL adenocarcinoma directly invaded the full thickness of the rectal wall (contiguous direct invasion), rather than representing a primary rectal carcinoma or hematogenous metastasis. Therefore, we recommended a comprehensive transabdominal staging surgery for the patient, including resection of the remaining right ovary, omentectomy, pelvic and para-aortic lymphadenectomy, as well as resection of peritoneal and intestinal lesions and multi-point biopsies. The patient and her family agreed to our surgical plan. Postoperative pathological examination revealed metastatic carcinoma in the right obturator lymph node and microscopic metastatic deposits.

**Figure 1:**
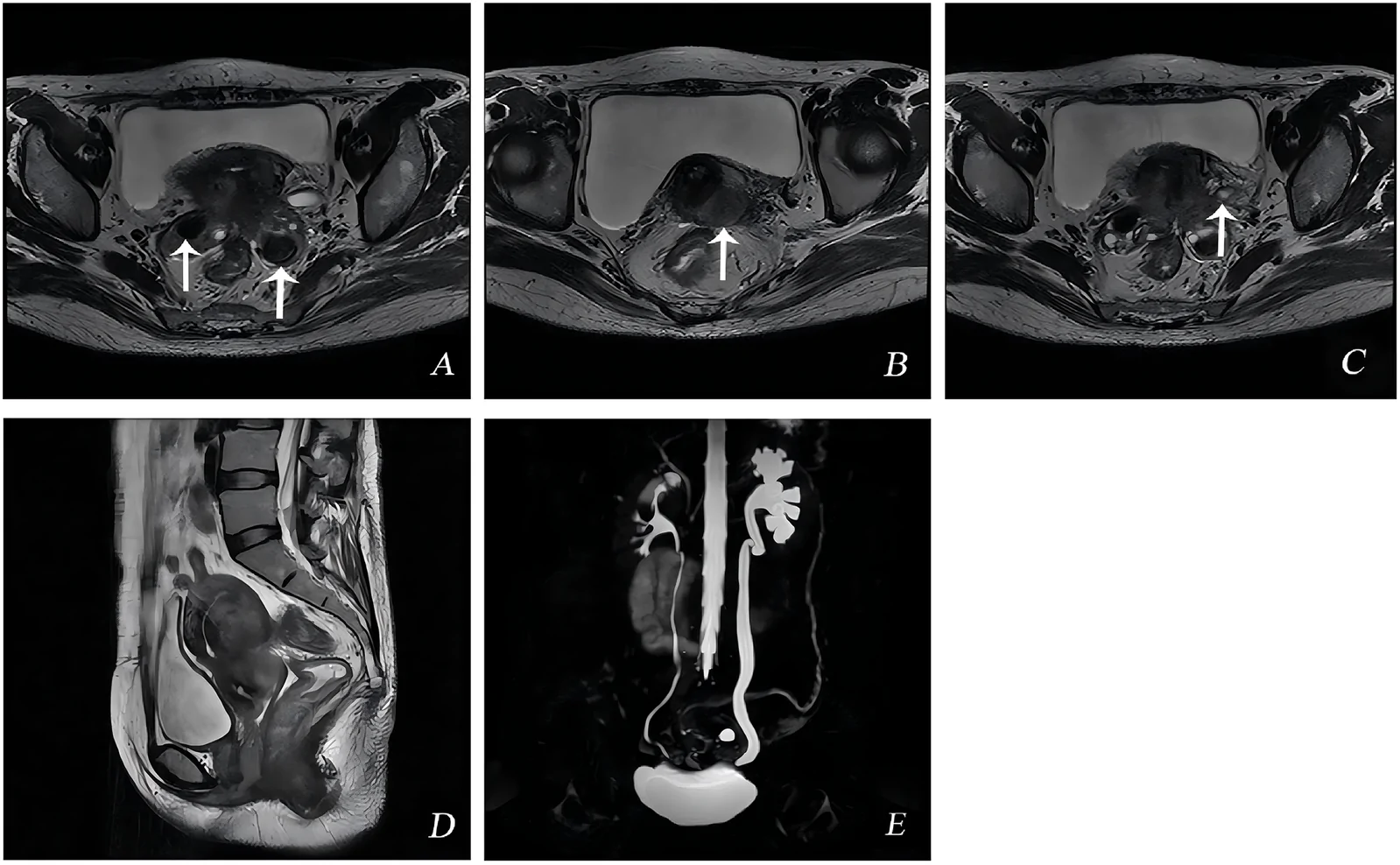
Pelvic magnetic resonance imaging (MRI). (**A**. top row, left) Axial T2-weighted imaging (Axial T2WI), at the level of the bilateral adnexa. The two white arrows indicate multiple endometriotic lesions in the bilateral adnexal regions, appearing as nodular lesions with heterogeneous signal and ill-defined borders. (**B**. top row, center) Axial T2-weighted imaging (Axial T2WI), at the level of the cervix. The white arrow indicates the main lesion in the left uterosacral ligament region, appearing as an infiltrative mass that is predominantly hypointense, with blurred margins and ill-defined demarcation from the surrounding tissues. (**C**. top row, right) Axial T2-weighted imaging (Axial T2WI), at the lower pelvis. The white arrow indicates the lesion infiltrating and encasing the distal left ureter, causing ureteral luminal narrowing. (**D**. bottom row, left) Sagittal T2-weighted imaging (Sagittal T2WI). This figure directly demonstrates the craniocaudal extent of the lesion: it extends superiorly to involve the lower uterine segment and cervix, extends anteriorly to infiltrate the posterior vaginal fornix, and lies posteriorly adjacent to the anterior rectal wall, clearly illustrating the multi-organ involvement characteristic of deep infiltrating endometriosis. (**E**. bottom row, right) Coronal MR urography (Coronal MRU). This figure clearly shows secondary dilatation and hydronephrosis of the middle and upper segments of the left ureter and of the left renal pelvis and calyces, directly related to the infiltrative narrowing of the ureter caused by the lesion below.

**Figure 2:**
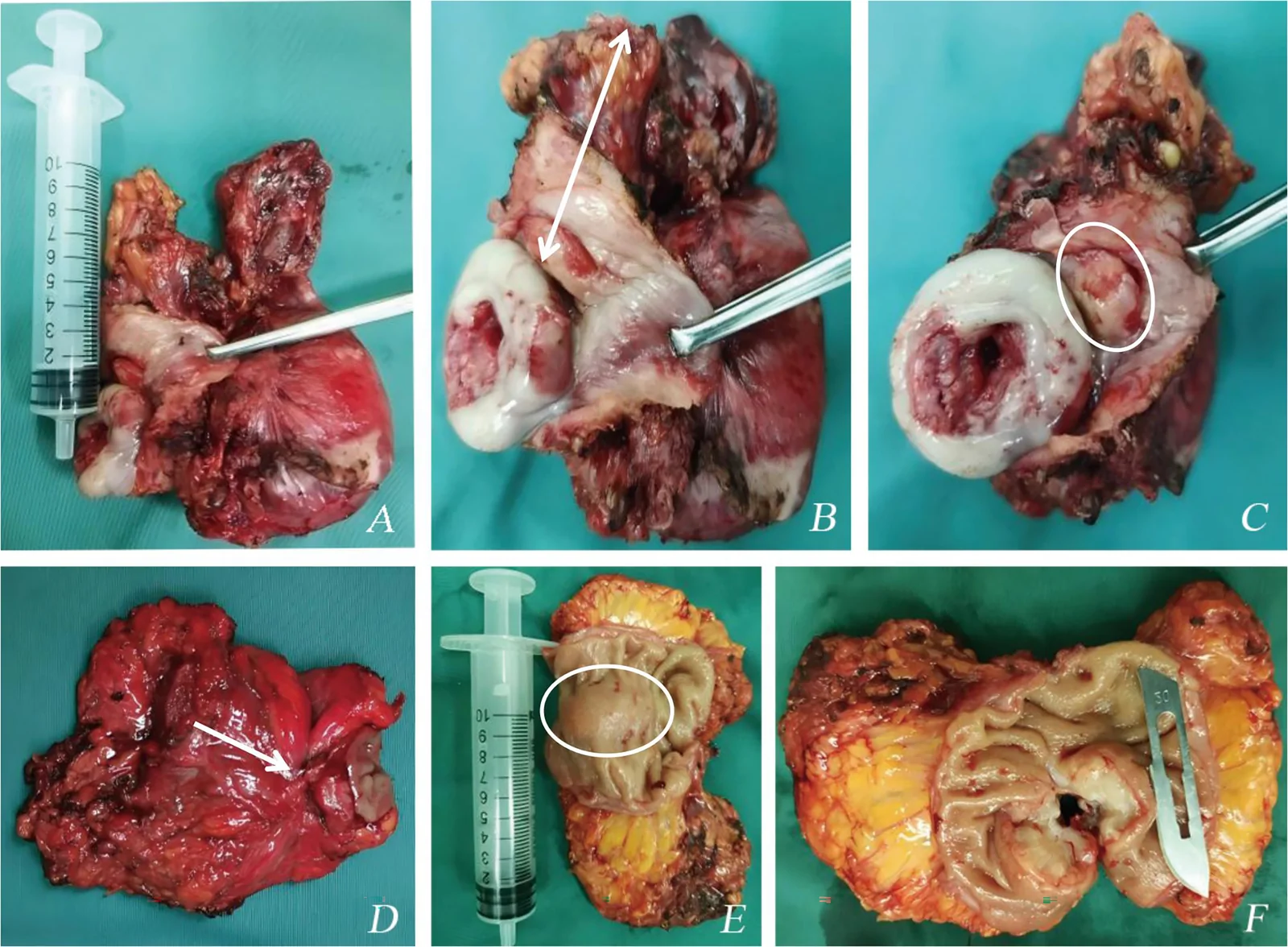
Postoperative specimens. **(A, B, C)** Gross specimens of deep infiltrating endometriosis (DIE) in the left uterosacral ligament, shown at different viewing angles and magnifications, primarily demonstrating the full-thickness infiltration of the lesion as it penetrates the vaginal wall. **(D)**Pelvic serosal surface view of the uterosacral ligament DIE lesion, demonstrating the border morphology of the lesion as it infiltrates and extends into the surrounding connective tissue.**(E, F)** Gross specimens of the rectal DIE lesion, shown as a mucosal surface view and a cross-sectional view of the bowel wall, respectively, demonstrating the pathological features of the lesion involving the full thickness of the bowel wall, accompanied by bowel contraction and narrowing.(White double-headed arrow in figure **(B)** Indicates the overall depth range of infiltration of the uterosacral ligament DIE lesion, directly illustrating the full-thickness infiltrative growth pattern of the lesion as it penetrates from the ligament serosa to the vaginal mucosa. White circle in figure **(C)** Circles the central indurated area of the uterosacral ligament lesion, which is the characteristic gross appearance formed by ectopic endometrial glands accompanied by extensive fibrous tissue proliferation; it is firm in consistency with ill-defined borders and represents the typical morphology of deep infiltrating endometriosis. White arrow in figure **(D)** Indicates the advancing infiltrative border of the lesion, which extends into the surrounding adipose connective tissue in a fibrous cord-like pattern, consistent with the invasive growth pattern of DIE. White circle in figure **(E)** Circles the elevated area of the DIE lesion on the rectal mucosal surface, where scattered dark red hemorrhagic spots are visible on the mucosal surface, corresponding to the cyclic bleeding of ectopic endometrial tissue.).

**Figure 3:**
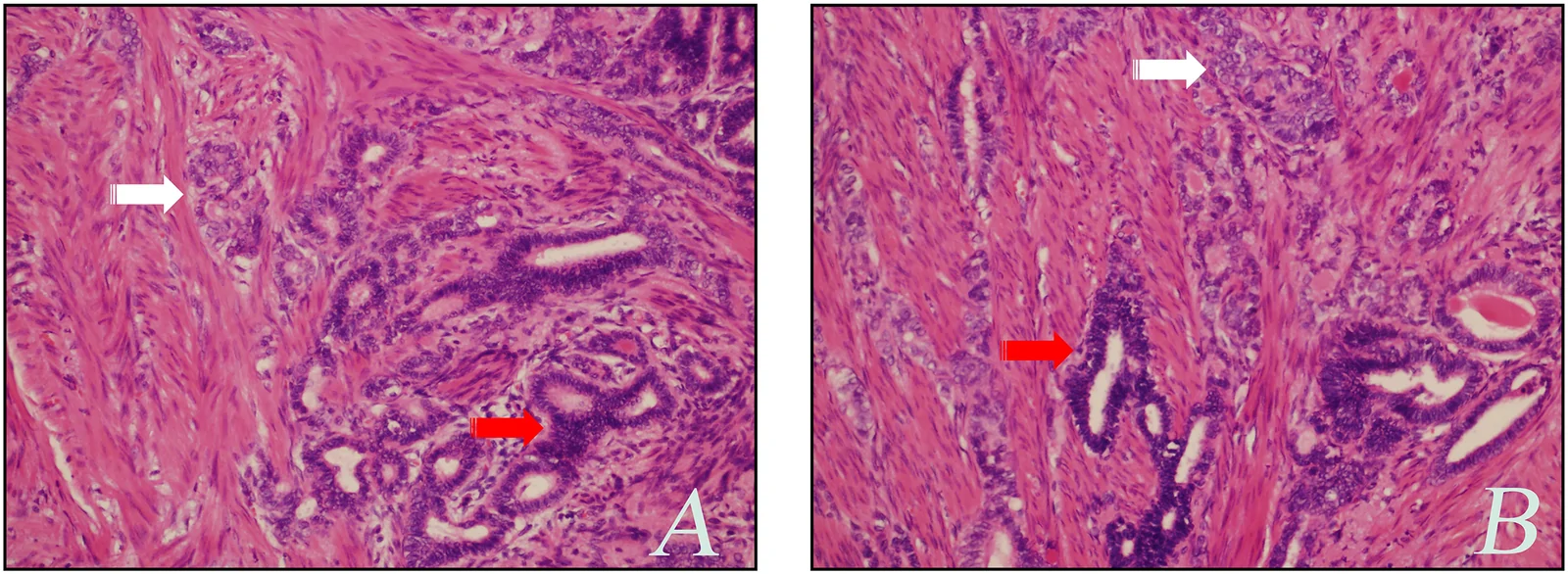
Postoperative pathology (uterosacral ligament, X100). **(A, B)** Coexistence of cancer cells (malignant transformed endometrial cells) and ectopic endometrial cells. White arrow indicates ectopic endometrial cells; red arrow indicates malignant transformed endometrial cells.

Reviewing the entire pathological findings:①Histopathological examination of the hysterectomy specimen confirmed that the native endometrium lining the uterine cavity showed benign histological features, with no atypical hyperplasia or invasive carcinoma. No primary malignant lesion was identified within the endometrial cavity, which directly rules out the possibility that a primary endometrial carcinoma had extended downward to involve the USL.②The atypical glands were distributed across two anatomically and pathologically distinct regions. Within the adenomyotic foci of the myometrium, scattered atypical glands were present but did not form confluent nests of invasive carcinoma, and they were anatomically discontinuous with the USL lesion. By contrast, the substance of the uterosacral ligament contained frankly invasive carcinomatous glands accompanied by benign ectopic endometrial stroma, representing the core focus of malignant transformation. This distribution pattern indicates that the carcinoma originated from ectopic endometrium within the USL, whereas the atypical glands in the adenomyosis represent either secondary involvement or coincident mild atypia rather than the primary tumor.③Anatomically, the bulk of the malignant lesion was initially confined to the left uterosacral ligament and exhibited infiltrative growth into adjacent structures: it penetrated the posterior vaginal wall, producing a vaginal vault lesion, and infiltrated the uterine serosa and superficial myometrium. No continuity was observed between the USL lesion and the adenomyotic foci in the deep myometrium. The presence of perineural invasion identified in the USL lesion further supports a pattern of local spread extending from the ligament into surrounding structures, rather than downward extension from the uterine cavity. The atypical glands observed in the cervix and vaginal wall were continuously spread from the USL lesion via the vaginal wall and parametrial tissues; no evidence of primary adenocarcinoma was identified in the native cervical mucosa, endocervical glands, or vaginal mucosal layer. ④Both ovaries were pathologically evaluated. The specimen from enucleation of the right ovarian endometriotic cyst showed a benign endometrioma with no evidence of malignant transformation. In the definitive surgery, the resected right ovary and left adnexa showed no primary ovarian carcinoma or metastatic involvement. These findings rule out ovarian endometrioid carcinoma as the primary source and further support that the USL lesion is an independent primary tumor arising from extraovarian endometriosis. Pathological examination confirmed that the rectal wall lesion was DIE (endometrioid-type glands and stroma within the bowel wall); the rectal mucosal layer was intact, with no adenomatous or malignant mucosal lesion.

She was diagnosed with stage IV disease according to the FIGO 2009 staging system (FIGO stage IV) pelvic endometriosis-associated malignancy. She received six cycles of adjuvant chemotherapy with paclitaxel and carboplatin administered every 3 weeks. During regular follow-up after chemotherapy, her dysmenorrhea and pelvic pain significantly improved, and her renal and ureteral function recovered well. There has been no evidence of tumor recurrence for more than 5 years since the surgery (tumor markers and imaging examinations are all normal). The patient has provided informed consent for publication of this case report.

## 3 Discussion

We report a rare case of extraovarian endometrioid adenocarcinoma arising from the uterosacral ligament in a 41-year-old woman, which originated from pelvic deep infiltrating endometriosis of the uterosacral ligament four years after a previous clinical diagnosis of adenomyosis.

Only around 1% of endometriosis cases experience malignant transformation, making it a rare event (4). Various reports have recorded instances of ovarian endometriosis resulting in malignant transformation. The incidence of ovarian cancer in women with ovarian endometriomas is approximately 0.7% (5). Reports indicate that about 0.2% of endometriosis cases in locations outside the ovaries, like the peritoneum and other extra-pelvic areas, undergo malignant transformation (6). Deep infiltrating endometriosis in the pelvis exhibits a notable predilection for the posterior pelvic compartment (7). In a study by Tarumi et al., 8 cases of malignant transformation from DIE involving the bladder were reviewed, though such occurrences are extremely rare (8). Endometrioid adenocarcinoma and clear cell adenocarcinoma are the main histological types of cancers found in both the rectovaginal septum and the bladder (8, 9). In comparison, the histological classifications for epithelial ovarian cancer connected to ovarian endometriomas comprise endometrioid, clear cell, and serous types.

The potential for cancer development may exist in endometriosis with unique molecular genetic properties. Mutations in the ARID1A gene have been reported in 30% of patients with endometrioid adenocarcinoma arising from ovarian endometriomas, and other genes, such as CTNNB1 and PTEN, may also be involved in this process (10, 11). Currently, research in this area has primarily focused on the analysis of patients with ovarian endometriomas, and further studies are required.

The patient in this study developed a malignancy originating from an endometriotic lesion in the uterosacral ligament. Malignant transformation at the uterosacral ligament is rarely reported.

The Sampson and Scott criteria represent the gold standard for the histopathological diagnosis of malignant transformation arising from endometriosis, and the present case fully satisfies these criteria.①Sampson criterion 1: An unequivocal endometriotic lesion is present adjacent to the tumor. In the uterosacral ligament specimen, typical benign ectopic endometrial glands and endometrial stroma were clearly visible in the tissue adjacent to the carcinoma, confirming the presence of a pre-existing deep infiltrating endometriosis at the tumor site.②Sampson criterion 2: The tumor is primary, exhibits endometrial histology, and metastasis has been excluded. Histologically, the tumor was a well-differentiated endometrioid adenocarcinoma (FIGO grade 1), the most common histological subtype of endometriosis-associated malignancy. Comprehensive staging surgery and systematic pathological examination excluded primary carcinomas of the uterus, ovaries, gastrointestinal tract, and other pelvic organs, confirming that the tumor was primary to the USL. The immunohistochemical findings further supported an endometrial origin.③Sampson criterion 3: Endometrial stroma is seen surrounding the epithelial glands under microscopy. In the benign endometriotic foci of the USL, typical endometrial stromal cells were seen encircling the ectopic glandular structures. In the areas of malignant transformation, residual endometrial stromal components were still present around the invasive carcinomatous glands, preserving the characteristic “stroma-surrounding-glands” histological pattern derived from endometriosis.④Sott’s additional criterion: Evidence of continuous transition from benign endometriosis to overt carcinoma is demonstrated. Histopathological sections (**Figure 3**) revealed a continuous morphological transition spectrum: benign ectopic endometrial glands → atypical hyperplastic glands → malignant carcinomatous glands, with no clear demarcation between them. This direct transitional evidence is the key morphological basis for confirming that the carcinoma arose from malignant transformation of pre-existing endometriosis, rather than representing an independent primary tumor or metastasis.

Taken together, all four diagnostic criteria are fulfilled, strongly supporting the conclusion that the endometrioid adenocarcinoma originated from malignant transformation of deep infiltrating endometriosis in the uterosacral ligament.

A case was documented by Yoshiaki et al. involving metachronous malignancy associated with endometriosis, developing from the uterosacral ligament, six years following treatment for a borderline ovarian tumor (12). In their report, the patient had initially undergone bilateral oophorectomy for a right borderline ovarian tumor. During follow-up, a suspected tumor recurrence was pathologically confirmed postoperatively as a malignancy arising from malignant transformation of endometriosis, located at the uterosacral ligament. In the present case, the patient presented with progressively worsening menstrual abdominal pain 8 years prior and was diagnosed with adenomyosis, receiving only conservative treatment. Lazzeri et al. have previously indicated that DIE is associated with 70% of adenomyosis cases (13), but the possibility of DIE was not recognized at that time. Five years prior, her symptoms worsened with the emergence of anal bulging pain and dyspareunia, which were still not adequately addressed. Subsequently, she developed lower abdominal pain accompanied by lumbosacral pain. A diagnostic laparoscopy at another hospital yielded a relatively clear diagnosis of pelvic DIE. Upon presentation at our hospital, further examinations were performed to assess the infiltration of surrounding tissues and organs by DIE lesions, in order to formulate an individualized surgical plan. Intraoperative exploration revealed no tissues with obvious malignant tendencies. After postoperative pathological examination ruled out primary tumors of the ovary and endometrium, the origin of the malignant tissue was traced back to ectopic endometrial tissue in the uterosacral ligament.

The preoperative pelvic MRI mainly showed the typical manifestations of deep infiltrating endometriosis (DIE): thickening of the uterosacral ligament, infiltrative growth of the lesion, and involvement of adjacent organs such as the vagina, rectum, and ureter. However, these findings of “infiltrative growth and multi-organ involvement” are also common features of severe benign DIE. No specific imaging signs suggestive of malignancy were present—such as extensive necrosis within the lesion, an exophytic irregular mass, marked high signal on diffusion-weighted imaging (DWI), or marked heterogeneous enhancement in the arterial phase. Therefore, it was difficult to directly suggest malignant transformation based on imaging alone before surgery. On the one hand, malignant transformation of endometriosis itself is extremely rare (approximately 0.5%–1%), and it is often hidden deep within benign DIE foci; its imaging features overlap substantially with the infiltrative pattern of benign DIE, which remains a general challenge in imaging diagnosis. On the other hand, this case was clinically highly suspected to be benign DIE before surgery, and the main purpose of the imaging examination was to assess the anatomical extent of the lesion and the degree of organ involvement so as to guide the surgical resection extent. The fact that functional sequences required for malignancy evaluation—such as multiphasic dynamic contrast-enhanced imaging and quantitative DWI analysis—were not specifically added also limited, to some extent, the recognition of early occult malignant transformation. This case also reminds clinicians that vigilance for malignant transformation should be maintained in DIE patients with rapidly progressive symptoms and a poor response to medical therapy; when necessary, functional imaging studies can be added to improve the preoperative detection rate of malignant transformation. **Figure 4** illustrates the origin, progression, and treatment strategy for the lesions in this patient.

**Figure 4:**
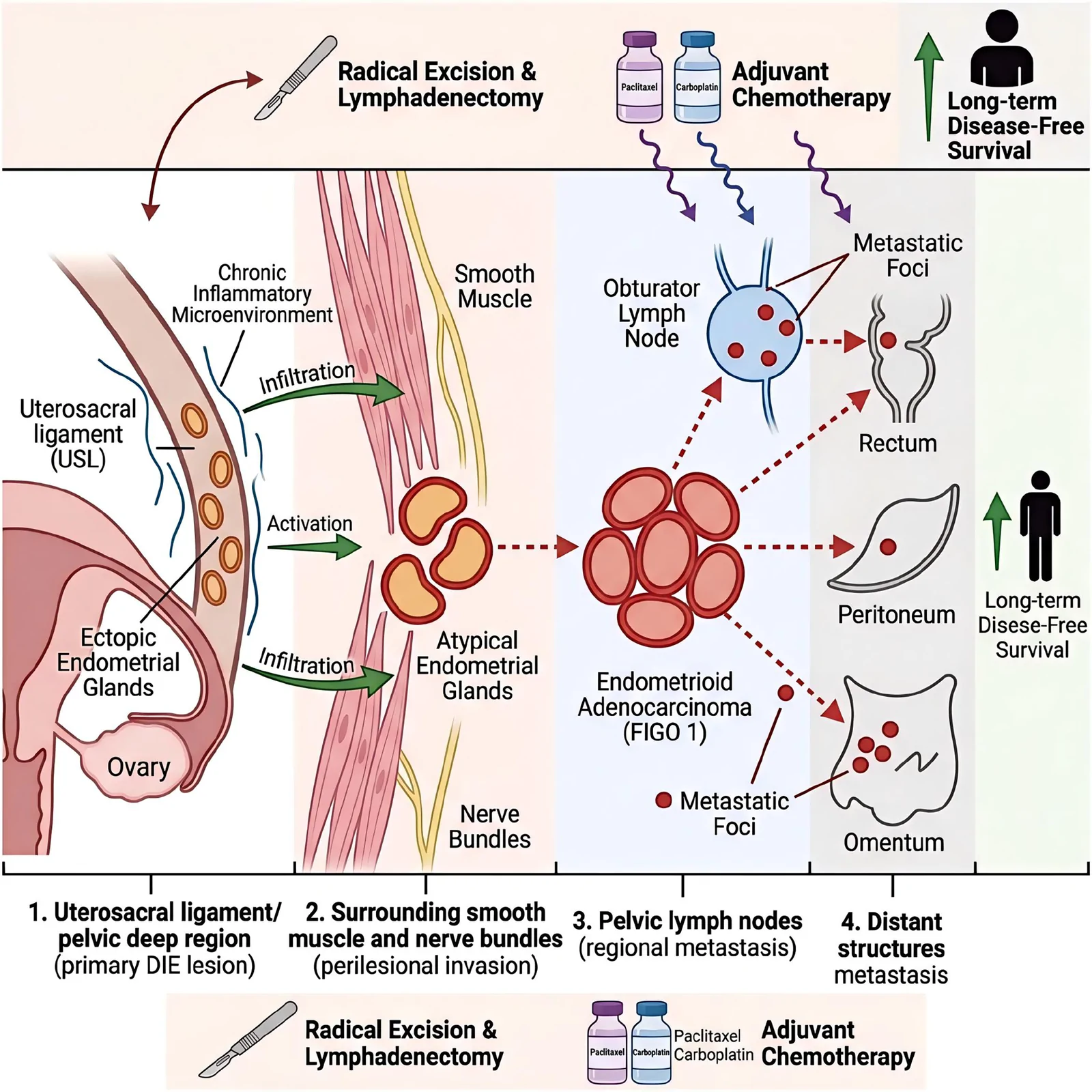
Origin, progression, and treatment strategy of the patient’s lesions.

Currently, there is no consensus on the treatment principles for extraovarian endometriosis-associated malignancy. Treatment primarily involves surgery, supplemented by hormone therapy, radiotherapy, and chemotherapy (14, 15). A previous case report described malignant transformation of DIE in the rectovaginal septum, where the patient underwent comprehensive staging surgery as supplemental treatment after pathological confirmation of malignancy, followed by adjuvant chemotherapy with paclitaxel and carboplatin (16). Considering DIE is a hormone-dependent disease, our patient similarly underwent comprehensive staging surgery, including resection of the remaining ovary, omentum, and pelvic and para-aortic lymph nodes. Postoperative pathology revealed metastatic carcinoma in the obturator lymph node, confirming the necessity and appropriateness of our secondary surgery. Referring to chemotherapy regimens for ovarian cancer, she was treated with carboplatin and paclitaxel (17). No recurrence has been observed during more than 5 years of follow-up.

Even though there seems to be a link between endometriosis and epithelial ovarian or endometrial cancers, endometriosis is not viewed as a precancerous condition, and screening is not advised. Typically, the European Society of Human Reproduction and Embryology (ESHRE) guidelines advise against surgically removing asymptomatic peritoneal endometriosis lesions (18). Currently, there is no evidence suggesting that removing ovarian endometriomas, peritoneal endometriosis lesions, or deep infiltrating endometriosis (DIE) lesions as a preventive measure can lower the risk of them becoming cancerous (19). In terms of treatment, several studies have recently explored the therapeutic efficacy of immune checkpoint inhibitors in patients with endometriosis and its associated malignancies. The results suggest that agents targeting PD-1/PD-L1/PD-L2 hold promise for conferring positive clinical benefits in this patient population (20–22).

In conclusion, we report a rare case of extraovarian endometrioid adenocarcinoma arising from the uterosacral ligament in a 41-year-old woman, that arose from pelvic deep infiltrating endometriosis of the uterosacral ligament, occurring four years after a previous clinical diagnosis of adenomyosis. For endometriosis presenting in atypical locations, with atypical symptoms, or with poor response to treatment, clinicians should maintain a high index of suspicion for the possibility of malignant transformation. For patients with endometriosis who present with typical clinical manifestations and no high-risk suspicious features, the routine diagnostic and therapeutic principles set forth in domestic and international guidelines should still be strictly followed, with lesion resection, preservation of function, and symptom control as the core goals. Routine extended resection according to malignancy standards is not recommended, so as to avoid subjecting the vast majority of benign patients to unnecessary surgical trauma and organ function damage for a malignant transformation event with an incidence of less than 1%.For patients with high-risk suspicious features of malignant transformation—including lesions at uncommon extraovarian sites such as the uterosacral ligament, vagina, or bladder; no symptom relief or even progressive worsening after standard medical therapy; mucosal ulceration or necrosis within the lesion; or an atypical infiltrative growth pattern on imaging—clinicians are advised to maintain a high index of suspicion, resect the lesion as completely as possible during surgery, and submit it routinely for intraoperative frozen section examination. If frozen pathology suggests malignant transformation or severe dysplasia, a comprehensive staging surgery should be completed according to the principles of malignancy management so as to safeguard the oncological prognosis. Applied to the present case, the patient presented with several suspicious features, including “DIE in the uterosacral ligament, poor response to medical therapy, and a posterior vaginal fornix ulcer.” The initial surgery achieved complete resection of the lesion according to DIE standards, and a comprehensive staging surgery was promptly added after postoperative pathology confirmed malignant transformation, ultimately resulting in a favorable long-term prognosis. This also confirms that, for suspicious cases, the risk-stratified strategy of “complete resection + intraoperative frozen section + extended surgery as needed” is a reasonable approach that balances surgical safety against oncological benefit. Given the potential for malignant transformation of endometriosis, particularly the deep infiltrating subtype, long-term follow-up is also essential. The patient remains free of recurrence for more than 5 years after surgery. However, as this is a single case, the relative contributions of surgical resection, tumor biology, and adjuvant chemotherapy to this favorable outcome cannot be distinguished; therefore, this good prognosis cannot serve as evidence of the effectiveness of the selected treatment and is provided for clinical reference only.

This study has the following limitations. First, due to the constraints of patient privacy protection and ethics approval, we were unable to perform supplementary immunohistochemical detection of key molecular markers such as ARID1A on the archived tissue specimens, and therefore could not further verify the clonal homology between the ectopic endometrial tissue and the carcinoma at the molecular genetic level. Second, this study is a single case report with a relatively limited follow-up duration; more case accumulation and long-term follow-up data are needed to further summarize the clinicopathological features and prognostic patterns of extraovarian DIE malignant transformation.

## Data Availability

The original contributions presented in the study are included in the article/**Supplementary Material**. Further inquiries can be directed to the corresponding author/s.
