# Supplementary material for: Malignant transformation of deep infiltrating endometriosis at the uterosacral ligament to endometrioid adenocarcinoma: a rare Case Report
Source: Front Oncol. 2026 Oct 14;16:1837994. doi: 10.3389/fonc.2026.1837994 (PMC13713408; doi:10.3389/fonc.2026.1837994)
Supplement: Supplementary file 1 [file Table1.docx]

Supplementary Material

# 1 Supplementary Figures

## 1.1 Supplementary Figure 1

**Supplementary Figure 1.** Findings of the posterior vaginal fornix under colposcopy.
(A, B, C) Patchy dark red nodular protrusions were visible in the posterior vaginal fornix from different angles, with an approximately 1 cm ulcerated surface on the lesion.

## 1.2 Supplementary Figure 2

**Supplementary Figure 2.** Findings of transvaginal ultrasound.
(A, B) Transvaginal ultrasound demonstrated hypoechoic lesions in the following anatomical compartments. (1) Posterior uterine wall: a 3.1 × 2.3 × 2.8 cm hypoechoic area in the myometrium with ill-defined borders, consistent with adenomyosis. (2) Posterior to the cervix (rectovaginal septum / uterosacral ligament region): a 2.5 × 2.4 × 2.7 cm hypoechoic mass with ill-defined borders, closely adherent to the rectal wall and poorly mobile, indicating deep infiltration into the rectovaginal septum and uterosacral ligament. (3) Posterior to the right ovary: a 2.2 × 1.1 cm crescent-shaped hypoechoic lesion with unclear boundaries, closely related to the bowel. Intraoperatively, these lesions were found to be densely adherent and fixed to the rectum and sigmoid colon, corroborating the ultrasound findings of deep infiltration and restricted mobility.

## 1.3 Supplementary Figure 3

**Supplementary Figure 3.** Radionuclide renogram

Early filling of the right kidney was observed in the arterial phase 4 seconds after visualization of the abdominal aorta, whereas blood perfusion of the left kidney was significantly reduced. The outline of the left kidney was indistinct, with sparse radioactive distribution. The right kidney was clearly visualized, with normal morphology and size, and normal processes of radioactive concentration and excretion. Bladder visualization was normal. The morphology of the right renogram curve showed clear delineation of segments a, b, and c, with normal renal index (RI), half-excretion time (C1/2), residual rate (C15/b), and peak time (Tb). The left renogram curve exhibited a low-level prolonged pattern, rendering the various indices uninterpretable. Glomerular filtration rate (GFR, units: ml/min/1.73㎡): Total GFR: 90.79, Left GFR: 19.73, Right GFR: 71.06. Findings indicated severe functional impairment of the left kidney, primarily manifested as decreased GFR; right kidney function was essentially normal, with normal GFR.

## 1.4 Supplementary Figure 4

**Supplementary Figure 4.** Endoscopic ultrasonography (EUS).
(A) Extraluminal irregular hypoechoic area. (B) The lesion locally infiltrating the muscularis propria of the intestinal wall.
